# A Simulation-based Quality Improvement Approach to Improve Pediatric Resident Competency with Required Procedures

**DOI:** 10.7759/cureus.1307

**Published:** 2017-06-03

**Authors:** Michelle Starr, Taylor Sawyer, Maya Jones, Maneesh Batra, Heather McPhillips

**Affiliations:** 1 Pediatrics, Seattle Children's Hospital; 2 Department of Pediatrics, University of Washington

**Keywords:** simulation, pediatric procedures, boot camp, resident training, acgme

## Abstract

Introduction: Pediatric residents report a lack of confidence and competence with procedural skills at graduation. Training programs could benefit from improved approaches to target these needs. Using the Institute for Healthcare Improvement (IHI) Model for Improvement and three Plan-Do-Study-Act (PDSA) cycles, we examined the impact of a procedure simulation boot camp on self-reported procedural confidence and competence as well as the longitudinal impacts of these sequential interventions on Accreditation Council for Graduate Medical Education (ACGME) Graduating Resident Survey (GRS) results.

Methods: Three rapid cycle interventions were performed in successive academic years. The interventions included 1) increased awareness of available procedural experiences, 2) institution of procedural educational conferences, and 3) implementation of a senior resident procedure boot camp. Senior resident self-reported procedural confidence was measured before and after the boot camp. Procedural competence was measured using the ACGME GRS.

Results: Thirty-two of 34 senior residents (94%) completed the 2016 ACGME GRS, similar to the response rates of 2014 (92%) and 2015 (94%), and 30 of 34 third-year residents participated in the procedure boot camp (88%). Resident confidence and competence with procedural skills improved after the institution of the quality improvement intervention. ACGME GRS-reported competency increased in bag and mask ventilation (77% to 94%), neonatal endotracheal intubation (39% to 69%), peripheral IV placement (10% to 50%), and umbilical catheter placement (35% to 53%).

Conclusion: A quality improvement intervention with three rapid PDSA cycles was successful in improving senior pediatric resident confidence and competence with ACGME required procedural skills.

## Introduction

The Accreditation Council for Graduate Medical Education (ACGME) Residency Review Committee for Pediatrics mandates that pediatric residents demonstrate procedural competence in 13 procedures (Table 1) [1].

**Table 1:** ACGME Required Procedures for Pediatric Residents ACGME: Accreditation Council for Graduate Medical Education

ACGME Required Procedures for Pediatric Residents
Bag mask ventilation
Bladder catheterization
Giving immunizations
Incision and drainage of abscess
Lumbar puncture
Neonatal endotracheal intubation
Peripheral intravenous catheter placement
Reduction of simple dislocation
Simple laceration repair
Simple removal of foreign body
Temporary splinting of fracture
Umbilical catheter placement
Venipuncture

However, numerous studies report that residents lack these skills. Recent studies have reported pediatric resident neonatal intubation success rates of only 20-26% [2-4]. A 2007 survey of pediatric program directors reported that fewer than 65% felt that all, or almost all, of their graduating residents were competent in the following procedures: peripheral intravenous (PIV) placement (48%), umbilical venous catheter (UVC) placement (57%), intubation (60%), and venipuncture (61%) [5].

Despite concerns regarding procedural competency for pediatric trainees, there is no agreement on methods to improve procedural training. Some programs have demonstrated that procedural simulation workshops increase resident self-reported confidence and competence based on measurements made immediately after the workshops [6]. However, no studies have assessed the impact of pediatric procedural education on self-reported competency as measured by the annual ACGME Graduation Resident Survey (GRS).

The objective of this study was to use quality improvement methodology to improve pediatric resident procedural competence, specifically focusing on self-reported procedural confidence and competence, the impact of a procedure simulation boot camp on self-reported procedural confidence and competence, and the longitudinal impacts of these sequential interventions on ACGME GRS results.

## Materials and methods

### Context

Seattle Children’s Hospital is a 300 bed, academic, free-standing children’s hospital with a 30-bed pediatric intensive care unit and a 26-bed neonatal intensive care unit. In addition, pediatric residents rotate through the University of Washington Neonatal and Newborn Services (2,000 deliveries per year). The pediatric residency program consists of approximately 112 total residents, 36 per year. Typically half of the graduates from this program pursue careers in general pediatrics or hospital medicine in the Pacific Northwest, and many graduates work in practices in small to mid-size cities without a nearby Children’s Hospital or ready access to pediatric specialists. This quality improvement (QI) effort was undertaken to address ACGME GRS scores for procedural competence that were below the national mean for two consecutive years. Program leadership also recognized that many of these procedural skills were necessary for optimally training the pediatric workforce for our region. The project was approved by Seattle Children’s Institutional Review Board.

### Increased awareness of procedural experiences – PDSA cycle 1

Before this intervention, residents received informal procedural training by supervisors often immediately before or during a procedure. Formal education was provided every two years in the Pediatric Advanced Life Support (PALS) course and once during the intern year in the Neonatal Resuscitation Program (NRP) course. During the July 2014 – June 2015 academic year, we focused on improving resident awareness of their procedural training. In coordination with the faculty-resident education committee, we worked to improve resident identification of existing procedural training during residency. Based on qualitative graduating resident feedback, we identified procedures that residents felt least competent with to improve our procedural education curriculum targeting these procedures.

### Procedural education curriculum – PDSA cycle 2

Our second PDSA cycle utilized resident feedback, as well as ACGME GRS results, to design a series of teaching conferences during the July 2015 – June 2016 academic year focusing on procedural skills that were easily taught in a large group setting. We held eight procedural lunchtime conferences covering splinting, laceration repair, and immunizations. Resident feedback on the content was sought after each conference and improvements were made on a monthly basis.

### Procedure simulation boot camp – PDSA cycle 3

Our third PDSA cycle was the implementation of a senior resident simulation boot camp in January 2016. Based on the ‘Learn-See-Practice-Prove-Do-Maintain’ pedagogy for procedural skills training, residents were asked to review preparatory materials, including written materials and instructional videos for each procedure, prior to boot camp [7]. During the half-day workshop, residents rotated in small groups (five to six per group) spending 40-45 minutes at each of the four stations listed in Table 2. Each station was run by faculty teaching procedures relevant to their field. Instructors reviewed the indications, contraindications, and risks of procedures and demonstrated the proper technique with high-fidelity patient simulators. Residents took turns performing each procedure under the direct supervision of the instructors, were given direct feedback, and repeated the procedure until they met competence criteria for that station.

**Table 2:** Boot Camp Procedure Stations IV: intravenous

Procedure Stations	Procedures Taught
Pediatric airway management	Bag-mask ventilation
	Laryngeal mask placement
	Endotracheal intubation
Neonatal airway management	Bag-mask ventilation
	Laryngeal mask placement
	Endotracheal intubation
Vascular access	Peripheral IV placement
	Intraosseous needle placement
Neonatal vascular access	Emergent umbilical vein catheterization

Residents completed two anonymous surveys, one immediately prior to boot camp and one immediately following the boot camp. In the pre-boot camp survey, residents were asked to indicate the number of times they had performed specific procedures during their residency, their confidence in performing each procedure, and to rate their self-percieved competence. In the post-boot camp survey, residents rated their confidence and their self-percieved competence in performing each procedure. Estimations of confidence and competence utilized a 5-point Likert-type scale (1 = "not confident" / "not competent" to 5 = "very confident" / "very competent").

### ACGME GRS

Graduating senior resident procedural competence was determined based on results of the ACGME GRS performed in the spring of each academic year. Competency was considered a response of “Agree” or “Strongly Agree” to the question “I feel well prepared to perform the following procedures without supervision.”

### Data analysis

Survey data were analyzed using matched variables and compared using paired t-tests. Differences were considered statistically significant if P < 0.05. ACGME survey data were evaluated using descriptive statistics, including means and standard deviations. Data analysis was performed using Microsoft Excel (XP) (Microsoft Corp., Redmond, WA) and Stata, version 12 (StataCorp, LLC, College Station, TX).

## Results

### Procedure simulation boot camp

Thirty of 34 third-year pediatric residents in our residency program participated in the procedure boot camp (88% participation rate). All residents who attended the boot camp completed pre-boot camp and post-boot camp surveys (100% completion rate). The estimated total numbers of procedures performed during residency are shown in Table 3. Even for procedures that were performed by all or most residents, the total number of times each procedure had been performed were low.

**Table 3:** Number of Procedures Performed by Pediatric Residents Involved in Boot Camp n: number; IV: intravenous

Procedure	Number of residents who performed the procedure at least once in clinical practice, n (%)	Number of procedures performed by each resident in clinical practice, range (mean)
Bag-mask ventilation	33 (97)	0 – 10 (3.7)
Neonatal endotracheal intubation	33 (97)	0 – 6 (2.8)
Pediatric endotracheal intubation	16 (47)	0 – 6 (2.3)
Peripheral IV placement	16 (47)	0 – 15 (5.7)
Intraosseous needle placement	2 (6)	0 – 3 (3.0)
Umbilical vein catheter placement	34 (100)	0 – 10 (3.4)
Emergent umbilical catheter placement	1 (3)	0 – 1 (1.0)

Resident self-assessed procedural confidence and competence are shown in Table 4. For each procedure, confidence increased by 8% to 48% (average: 30%) and competence increased by 17% to 49% (average: 33%). Differences between pre-boot camp and post-boot camp confidence for all procedures (with the exception of bag-mask ventilation) and between pre-boot camp and post-boot camp competence for all procedures assessed were statistically significant (Table 4).

**Table 4:** Resident Pre-boot Camp and Post-boot Camp Confidence and Competence IV: intravenous

	Confidence			Competence		
	Pre	Post	P	Pre	Post	P
Bag-mask ventilation, mean	4.2	4.5	0.076	3.8	4.4	< 0.001
Neonatal endotracheal intubation	2.5	3.5	< 0.001	2.5	3.1	0.015
Pediatric endotracheal intubation	2.1	3.1	< 0.001	2.0	2.9	< 0.001
Peripheral IV placement	3.2	3.8	0.013	2.9	3.5	0.028
Intraosseous needle placement	2.9	4.0	< 0.001	2.6	3.8	< 0.001
Emergent umbilical vein catheter placement	2.9	3.8	< 0.001	2.7	3.7	0.002

### ACGME GRS results

Thirty-two of 34 senior residents (94%) completed the 2016 ACGME GRS, which was similar to the response rates from our program to the GRS in 2014 (92%) and 2015 (94%). Procedures, which were taught in a large group setting in the Procedural Educational Curriculum (PDSA Cycle 2), demonstrated a variable change in resident competency. Competency was increased for immunization administration (26% to 69%) and laceration repairs (87% to 94%) but unchanged for splinting (74% to 75%).

**Figure 1:**
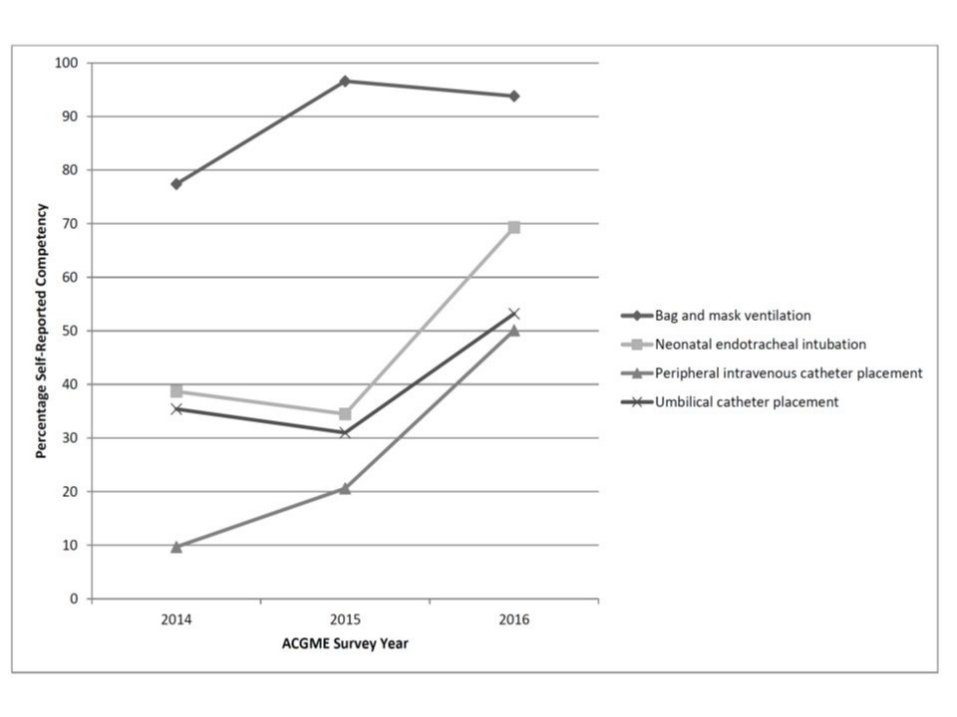
Resident Procedural Competency as Assessed by ACGME Graduating Resident Survey ACGME: Accreditation Council for Graduate Medical Education

Four ACGME required procedures were taught during the half-day boot camp (PDSA cycle 3). For all, the percentage of graduating residents reporting competence increased from 2014 to 2016 for bag and mask ventilation (reported competence increased from 77% to 94%), neonatal endotracheal intubation (39% to 69%), peripheral intravenous catheter placement (10% to 50%), and umbilical catheter placement (35% to 53%) (Figure 1).

Procedures which were not addressed with the second or third PDSA cycles demonstrated mixed results for resident competency, with no change for lumbar puncture (97%), decreases in self-reported competency for reduction of dislocations (81% to 69%), and foreign body removals (87% to 81%), and increases in resident competency for delivery room resuscitation (87% to 100%), bladder catheterization (16% to 31%), and incision and drainage of an abscess (71% to 87%).

## Discussion

Our study found that a quality improvement intervention enhanced procedural confidence and competence among graduating pediatric residents. We found a positive impact of our procedural simulation training on ACGME procedural competency scores, with the most significant changes in procedural competence seen in skills taught in a simulation-based procedural boot camp (PDSA cycle 3). We believe these results reflect the impact of the boot camp training and not the result of general trends in resident training or observation biases. This lends validity to the impact of the simulation-based boot camp training as an effective methodology.

As residents’ experiential training faces growing time restrictions, the implementation of adjuvant educational methods is critical. The lack of procedural competence seems to be a growing concern and may be a reflection of limited opportunities [7]. As we have demonstrated, one promising educational modality is the use of simulation training, which can be used to provide realistic patient procedural training [8]. Some studies have suggested that simulation-based training is superior when compared with traditional experiential training in gaining competency with specific skills [9]. In one study, pediatric residents who received simulation-based education were more successful in peripheral intravenous catheter placement and lumbar puncture than those taught with other methods [10]. Modern procedural training paradigms leverage simulation as the primary educational method [11]. Indeed, the standard practice of “see one, do one, teach one” may eventually evolve into “see one, simulate many, do one competently, and teach everyone” [12].

We showed that participation in a simulation boot camp improved resident self-reported procedural confidence and competence. Our results support the results of other studies in this area. Cohen, et al. reported that a simulation-based mastery learning boot camp for internal medicine interns improved clinical skills scores on five parts of a clinical skills examination as compared to historical controls [13]. Krajewski, et al. found that a two-month boot camp curriculum consisting of knowledge-based and procedural skills sessions, completion of web-based self-study modules, and standardized patient clinical skills assessments resulted in better patient care, procedural skills, and self-confidence compared with the previous year's interns, as judged by the teaching faculty [14].

The next steps of this QI project include expanding the procedural education curriculum to include more procedures and more sessions. We are planning for monthly large group procedure teaching sessions in the July 2016 - June 2017 academic year, as well as increasing participation in the simulation-based procedural boot camp by including second-year residents. We plan to continue frequent PDSA cycles to optimize resident procedure training, with the goal of graduating procedurally competent residents.

Our educational intervention had several limitations. First, as residents were aware of our ongoing efforts to improve procedural competence and that procedure competency was an important issue to the residency program, there may have been an element of reporting bias. Secondly, we chose to assess both ‘confidence’, defined as feeling well prepared to perform the procedure, as well as ‘competence’, defined as the ability to perform a procedure correctly without direct supervision. Previous research into procedural training has shown that residents may be competent without reporting confidence in their procedural ability [6]. Conversely, other research has shown that confidence with a procedure does not correlate with procedural competency [15-16]. This effect is particularly noted when there is limited opportunity to perform the procedure clinically [17]. Third, resident self-reported confidence and competence were measured immediately after the procedure boot camp, and therefore, it is not clear if these improvements persisted over time. However, some conclusions can be drawn from the ACGME GRS as this was performed approximately four months after the procedure boot camp and may demonstrate retention of competence. Finally, we did not track translational outcomes from the procedural skills training. Therefore, we cannot comment on the impact of the interventions on the actual clinical skill and competency of our residents.

## Conclusions

The lack of pediatric resident procedural competence is a growing concern, both at our institution and nationwide, as residents perform fewer procedures in the clinical setting. A quality improvement educational intervention with several PDSA cycles was successful at improving resident reported procedural competence. Procedural simulation training, which included large group education sessions and targeted boot camps, was successful in increasing graduating resident reported procedural competence.
